# Supplementary material for: Machine Learning Accelerates Crystallization for Structure Determination
Source: Angew Chem Int Ed Engl. 2026 May 4;65(25):e1218503. doi: 10.1002/anie.1218503 (PMC13266958; doi:10.1002/anie.1218503)

checkCIF/PLATON report

Structure factors have been supplied for datablock(s) lcz110

THIS REPORT IS FOR GUIDANCE ONLY. IF USED AS PART OF A REVIEW PROCEDURE FOR PUBLICATION, IT SHOULD NOT REPLACE THE EXPERTISE OF AN EXPERIENCED CRYSTALLOGRAPHIC REFEREE.

No syntax errors found. CIF dictionary Interpreting this report

Datablock: lcz110

Bond precision:	C-C = 0.0348 A		Wavelength=1.54184
Cell:	a=23.4213(8)	b=13.5037(3)	c=28.3748(11)
	alpha=90	beta=104.746(4)	gamma=90
Temperature:	100 K		

	Calculated	Reported
Volume	8678.6(5)	8678.6(5)
Space group	C 2	C 1 2 1
Hall group	C 2y	C 2y
Moiety formula	C138 H106 Ag12 F72 N26 O28	2(C15 H3 Ag3 F18 N6), C39 H47 N O14
Sum formula	C138 H106 Ag12 F72 N26 O28	C69 H53 Ag6 F36 N13 O14
Mr	5238.94	2619.46
Dx,g cm-3	2.005	2.005
Z	2	4
Mu (mm-1)	11.978	11.977
F000	5104.0	5104.0
F000'	5127.61	
h,k,lmax	28,16,35	28,16,34
Nref	17261[9019]	14916
Tmin,Tmax	0.150,0.187	0.716,1.000
Tmin'	0.042	

Correction method= # Reported T Limits: Tmin=0.716 Tmax=1.000
AbsCorr = MULTI-SCAN

```
Data completeness= 1.65/0.86       Theta(max)= 72.656

R(reflections)= 0.0765( 12688)                 wR2(reflections)=
                                                0.1935( 14916)
S = 1.042                 Npar= 1231
```

The following ALERTS were generated. Each ALERT has the format
test-name_ALERT_alert-type_alert-level.
Click on the hyperlinks for more details of the test.

Alert level B

```
PLAT342_ALERT_3_B Low Bond Precision on  C-C Bonds ..............    0.03483 Ang.
```

Author Response: Disordered structure.

Alert level C

```
PLAT041_ALERT_1_C Calc. and Reported SumFormula    Strings  Differ     Please Check
              Calc: C138 H106 Ag12 F72 N26 O28
              Rep.: C69 H53 Ag6 F36 N13 O14
PLAT042_ALERT_1_C Calc. and Reported MoietyFormula Strings  Differ     Please Check
              Calc: C138 H106 Ag12 F72 N26 O28
              Rep.: 2(C15 H3 Ag3 F18 N6), C39 H47 N O14
PLAT090_ALERT_3_C Poor Data / Parameter Ratio (Zmax > 18) ........       6.99 Note
PLAT213_ALERT_2_C Atom N031            has ADP max/min Ratio .....        3.6 prolat
PLAT213_ALERT_2_C Atom C022            has ADP max/min Ratio .....        3.1 oblate
PLAT213_ALERT_2_C Atom C39             has ADP max/min Ratio .....        4.0 prolat
PLAT220_ALERT_2_C NonSolvent   Resd 1  C   Ueq(max)/Ueq(min) Range        4.7 Ratio
PLAT220_ALERT_2_C NonSolvent   Resd 1 Ag   Ueq(max)/Ueq(min) Range        4.1 Ratio
PLAT220_ALERT_2_C NonSolvent   Resd 1  F   Ueq(max)/Ueq(min) Range        5.0 Ratio
PLAT220_ALERT_2_C NonSolvent   Resd 1  N   Ueq(max)/Ueq(min) Range        4.1 Ratio
PLAT220_ALERT_2_C NonSolvent   Resd 1  O   Ueq(max)/Ueq(min) Range        3.6 Ratio
PLAT230_ALERT_2_C Hirshfeld Test Diff for    F03F     --C87       .       5.3 s.u.
PLAT230_ALERT_2_C Hirshfeld Test Diff for    C02I     --C038      .       6.4 s.u.
PLAT234_ALERT_4_C Large Hirshfeld Difference Ag05     --O3        .      0.24 Ang.
PLAT234_ALERT_4_C Large Hirshfeld Difference F007     --C01B      .      0.18 Ang.
PLAT234_ALERT_4_C Large Hirshfeld Difference F00F     --C01Z      .      0.17 Ang.
PLAT234_ALERT_4_C Large Hirshfeld Difference F00P     --C02Z      .      0.22 Ang.
PLAT234_ALERT_4_C Large Hirshfeld Difference F00T     --C01X      .      0.18 Ang.
PLAT234_ALERT_4_C Large Hirshfeld Difference O00X     --C025      .      0.18 Ang.
PLAT234_ALERT_4_C Large Hirshfeld Difference O01F     --C02P      .      0.24 Ang.
PLAT234_ALERT_4_C Large Hirshfeld Difference N00E     --C01J      .      0.17 Ang.
PLAT234_ALERT_4_C Large Hirshfeld Difference N00V     --C015      .      0.18 Ang.
PLAT234_ALERT_4_C Large Hirshfeld Difference C019     --C02E      .      0.20 Ang.
PLAT241_ALERT_2_C High   'MainMol' Ueq as Compared to Neighbors of       Ag05 Check
PLAT241_ALERT_2_C High   'MainMol' Ueq as Compared to Neighbors of       Ag06 Check
PLAT241_ALERT_2_C High   'MainMol' Ueq as Compared to Neighbors of         O3 Check
PLAT241_ALERT_2_C High   'MainMol' Ueq as Compared to Neighbors of       C034 Check
PLAT241_ALERT_2_C High   'MainMol' Ueq as Compared to Neighbors of       C038 Check
PLAT242_ALERT_2_C Low    'MainMol' Ueq as Compared to Neighbors of       O01F Check
PLAT242_ALERT_2_C Low    'MainMol' Ueq as Compared to Neighbors of       C02I Check
```

```
PLAT242_ALERT_2_C Low    'MainMol' Ueq as Compared to Neighbors of       C033 Check
PLAT362_ALERT_2_C Short  C(sp3)-C(sp2) Bond  C033     - C87      .       1.39 Ang.
PLAT906_ALERT_3_C Large K Value in the Analysis of Variance ......      3.407 Check
PLAT911_ALERT_3_C Missing FCF Refl Between Thmin & STh/L=    0.600         79 Report
                0 16  0,   1 15  0,   2 16  0,   4 16  0,  -4 16  1,  -3 15  1,
               -2 16  1,  -1 15  1,   0 16  1,   1 15  1,   2 16  1,   4 16  1,
               -4 16  2,  -3 15  2,  -2 16  2,  -1 15  2,   0 16  2,   1 15  2,
                2 16  2,  -4 16  3,  -3 15  3,  -2 16  3,  -1 15  3,   0 16  3,
                2 16  3,   5 13  3,  10 12  3,  -3 15  4,  -2 16  4,  -1 15  4,
                   (   49 More Missing: see the .ckf listing file)
PLAT915_ALERT_3_C No Flack x Check Done: Low Friedel Pair Coverage         77 %
PLAT977_ALERT_2_C Check Negative Difference Density on H5A       .      -0.35 eA-3
```

Alert level G

```
PLAT002_ALERT_2_G Number of Distance or Angle Restraints on AtSite          3 Note
PLAT003_ALERT_2_G Number of Uiso or U(i,j)  Restrained non-H-Atoms          6 Report
PLAT007_ALERT_5_G Number of Unrefined Donor-H Atoms .............           4 Report
              H00C  H00H  H016  H01H
PLAT045_ALERT_1_G Calculated and Reported Z Differ by a Factor ...      0.500 Check
PLAT083_ALERT_2_G SHELXL Second Parameter in WGHT  Unusually Large     121.21 Why ?
PLAT171_ALERT_4_G The CIF-Embedded .res File Contains EADP Records          4 Report
PLAT172_ALERT_4_G The CIF-Embedded .res File Contains DFIX Records          2 Report
PLAT186_ALERT_4_G The CIF-Embedded .res File Contains ISOR Records          4 Report
PLAT242_ALERT_2_G Low    'MainMol' Ueq as Compared to Neighbors of         C4 Check
PLAT242_ALERT_2_G Low    'MainMol' Ueq as Compared to Neighbors of        C11 Check
PLAT242_ALERT_2_G Low    'MainMol' Ueq as Compared to Neighbors of       C01P Check
PLAT242_ALERT_2_G Low    'MainMol' Ueq as Compared to Neighbors of       C01X Check
PLAT242_ALERT_2_G Low    'MainMol' Ueq as Compared to Neighbors of       C01Z Check
PLAT242_ALERT_2_G Low    'MainMol' Ueq as Compared to Neighbors of       C022 Check
PLAT242_ALERT_2_G Low    'MainMol' Ueq as Compared to Neighbors of       C03A Check
PLAT242_ALERT_2_G Low    'MainMol' Ueq as Compared to Neighbors of       C03J Check
PLAT242_ALERT_2_G Low    'MainMol' Ueq as Compared to Neighbors of       C03L Check
PLAT242_ALERT_2_G Low    'MainMol' Ueq as Compared to Neighbors of        C87 Check
PLAT343_ALERT_2_G Unusual sp?      Angle Range in Main Residue for        C15 Check
PLAT367_ALERT_2_G Long?  C(sp?)-C(sp?) Bond  C5       - C15      .       1.52 Ang.
PLAT398_ALERT_2_G Deviating  C-O-C   Angle From 120 for O1       .      107.3 Degree
PLAT398_ALERT_2_G Deviating  C-O-C   Angle From 120 for O2       .      132.8 Degree
PLAT398_ALERT_2_G Deviating  C-O-C   Angle From 120 for O00W     .      109.3 Degree
PLAT434_ALERT_2_G Short Inter HL..HL Contact F00Q     ..F03N     .       2.84 Ang.
                                                  -x,y,1-z  =      2_556 Check
PLAT720_ALERT_4_G Number of Unusual/Non-Standard Labels ..........        176 Note
              Ag01    Ag02    Ag03    Ag04    Ag05    Ag06    F007    F008
              F009    F00A    F00B    O00C    H00C    F00D    N00E    F00F
              F00G    O00H    H00H    O00I    F00J    F00K    F00L    F00M
              F00N    F00O    F00P    F00Q    C00R    H00R    F00S    F00T
              N00U    N00V    O00W    O00X    N00Y    N00Z    F011    O012
              O013    O014    C015    O016    H016    C017    H017    C018
              C019    C01A    C01B    C01C    H01D    C01D    O01E    O01F
              N01H    H01H    C01I    C01J    C01K    H01K    C01L    H01L
              C01M    F01N    C01O    C01P    C01Q    C01R    H01R    C01S
              H01S    C01T    H01A    H01B    H01C    F01U    C01V    H01V
              C01W    H01W    C01X    N01Y    C01Z    F020    C021    C022
              F024    C025    C026    H026    C027    F028    C029    C02A
              H02A    F02B    C02C    H02B    H02C    H02D    C02D    H02E
              C02E    F02F    C02G    H02F    H02G    H02H    N02H    C02I
              C02J    H02I    H02J    H02K    N02K    N02L    C02M    H02M
```

```
              C02N    H02S    C02O    C02P    F02Q    C02R    H02L    H02N
              H02O    C02S    C02T    H02P    H02Q    H02R    C02U    H02U
              F02V    C02W    H02W    N02X    C02Y    H02Y    C02Z    C030
              H03A    H03B    H03C    N031    C032    H03D    H03E    H03F
              C033    C034    H034    F035    C036    F037    C038    F039
              C03A    C03B    H03G    H03H    H03I    F03C    C03D    F03E
              F03F    C03G    H03J    C03I    C03J    F03K    C03L    F03N
PLAT791_ALERT_4_G Model has Chirality at C01K       (Sohncke SpGr)          S Verify
PLAT791_ALERT_4_G Model has Chirality at C01L       (Sohncke SpGr)          S Verify
PLAT791_ALERT_4_G Model has Chirality at C01R       (Sohncke SpGr)          S Verify
PLAT791_ALERT_4_G Model has Chirality at C01S       (Sohncke SpGr)          R Verify
PLAT791_ALERT_4_G Model has Chirality at C01W       (Sohncke SpGr)          S Verify
PLAT791_ALERT_4_G Model has Chirality at C021       (Sohncke SpGr)          S Verify
PLAT791_ALERT_4_G Model has Chirality at C026       (Sohncke SpGr)          S Verify
PLAT791_ALERT_4_G Model has Chirality at C02P       (Sohncke SpGr)          S Verify
PLAT791_ALERT_4_G Model has Chirality at C02W       (Sohncke SpGr)          S Verify
PLAT791_ALERT_4_G Model has Chirality at C02Y       (Sohncke SpGr)          R Verify
PLAT860_ALERT_3_G Number of Least-Squares Restraints ............         39 Note
PLAT883_ALERT_1_G Absent Datum for _atom_sites_solution_primary ..     Please Do !
PLAT910_ALERT_3_G Missing FCF Reflection(s) Below Theta(Min)[Deg]=       3.81 Note
                0  0  1,   0  0  2,
PLAT912_ALERT_4_G Missing # of FCF Reflections Above STh/L=  0.600        326 Note
PLAT941_ALERT_3_G Average HKL Measurement Multiplicity ..........        4.5 Low
PLAT969_ALERT_5_G The 'Henn et al.' R-Factor-gap value ..........      3.024 Note
              Predicted wR2: Based on SigI**2  6.40 or SHELX Weight 18.57
PLAT978_ALERT_2_G Number C-C Bonds with Positive Residual Density.          0 Info
```

```
   0 ALERT level A = Most likely a serious problem - resolve or explain
   1 ALERT level B = A potentially serious problem, consider carefully
  36 ALERT level C = Check. Ensure it is not caused by an omission or oversight
  42 ALERT level G = General information/check it is not something unexpected

   4 ALERT type 1 CIF construction/syntax error, inconsistent or missing data
  40 ALERT type 2 Indicator that the structure model may be wrong or deficient
   8 ALERT type 3 Indicator that the structure quality may be low
  25 ALERT type 4 Improvement, methodology, query or suggestion
   2 ALERT type 5 Informative message, check
```

Validation response form

Please find below a validation response form (VRF) that can be filled in and pasted into your CIF.

```
# start Validation Reply Form
_vrf_PLAT041_lcz110
;
PROBLEM: Calc. and Reported SumFormula    Strings  Differ     Please Check
RESPONSE: ...
;
_vrf_PLAT042_lcz110
;
PROBLEM: Calc. and Reported MoietyFormula Strings  Differ     Please Check
RESPONSE: ...
;
_vrf_PLAT090_lcz110
;
```

```
PROBLEM: Poor Data / Parameter Ratio (Zmax > 18) ........       6.99 Note
RESPONSE: ...
;
_vrf_PLAT213_lcz110
;
PROBLEM: Atom N031            has ADP max/min Ratio .....        3.6 prolat
RESPONSE: ...
;
_vrf_PLAT220_lcz110
;
PROBLEM: NonSolvent   Resd 1  C   Ueq(max)/Ueq(min) Range        4.7 Ratio
RESPONSE: ...
;
_vrf_PLAT230_lcz110
;
PROBLEM: Hirshfeld Test Diff for    F03F     --C87      .        5.3 s.u.
RESPONSE: ...
;
_vrf_PLAT234_lcz110
;
PROBLEM: Large Hirshfeld Difference Ag05     --O3       .       0.24 Ang.
RESPONSE: ...
;
_vrf_PLAT241_lcz110
;
PROBLEM: High   'MainMol' Ueq as Compared to Neighbors of       Ag05 Check
RESPONSE: ...
;
_vrf_PLAT242_lcz110
;
PROBLEM: Low    'MainMol' Ueq as Compared to Neighbors of       O01F Check
RESPONSE: ...
;
_vrf_PLAT362_lcz110
;
PROBLEM: Short  C(sp3)-C(sp2) Bond  C033     - C87      .       1.39 Ang.
RESPONSE: ...
;
_vrf_PLAT906_lcz110
;
PROBLEM: Large K Value in the Analysis of Variance ......      3.407 Check
RESPONSE: ...
;
_vrf_PLAT911_lcz110
;
PROBLEM: Missing FCF Refl Between Thmin & STh/L=    0.600         79 Report
RESPONSE: ...
;
_vrf_PLAT915_lcz110
;
PROBLEM: No Flack x Check Done: Low Friedel Pair Coverage         77 %
RESPONSE: ...
;
_vrf_PLAT977_lcz110
;
```

```
PROBLEM: Check Negative Difference Density on H5A       .      -0.35 eA-3
RESPONSE: ...
;
# end Validation Reply Form
```

It is advisable to attempt to resolve as many as possible of the alerts in all categories. Often the minor alerts point to easily fixed oversights, errors and omissions in your CIF or refinement strategy, so attention to these fine details can be worthwhile. It is up to the individual to critically assess their own results and, if necessary, seek expert advice.

PLATON version of 17/09/2025; check.def file version of 17/09/2025

---

duplicate check

No duplication found

Datablock lcz110 - ellipsoid plot

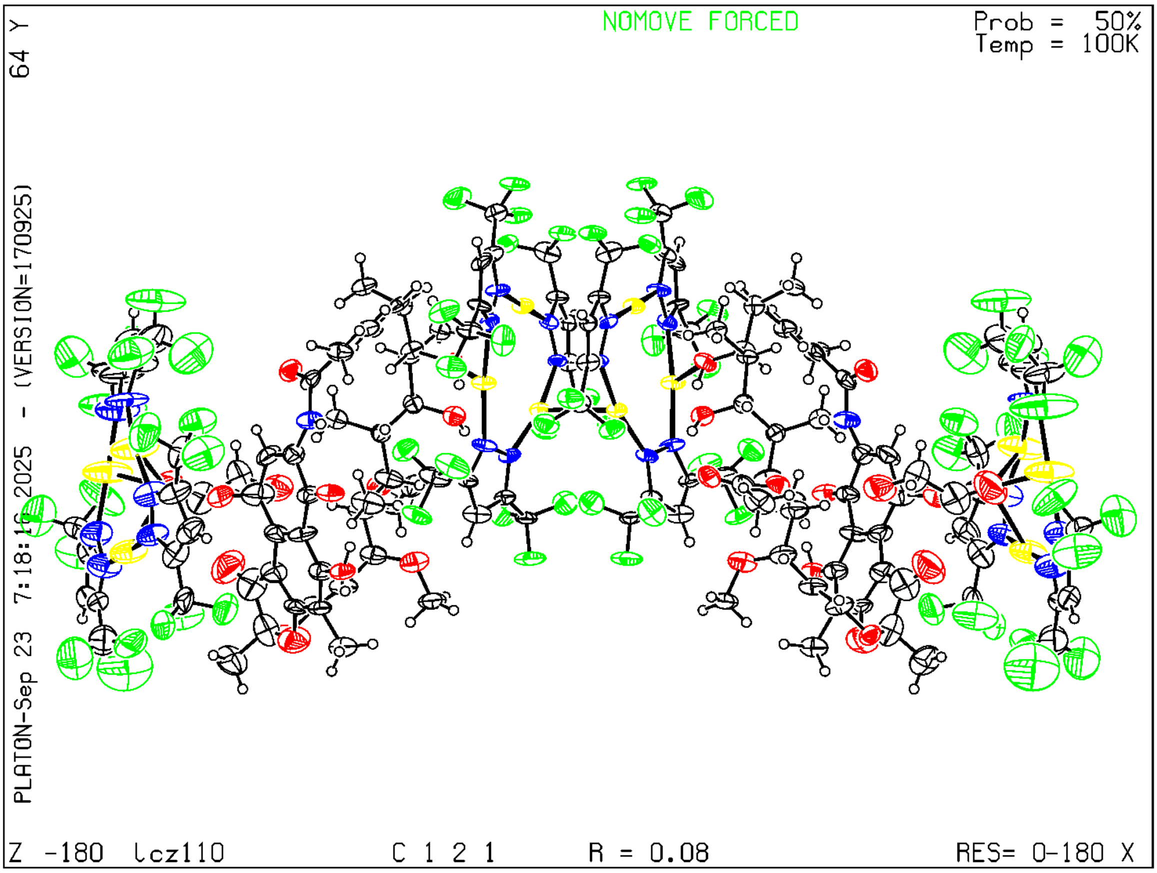

Supplement: Supplementary file 2 — Supporting File 2: anie72462‐sup‐0002‐DataFile.zip. [file ANIE-65-e1218503-s002.zip › supplementary_materials_crystallographic_data submit.zip!/cif_and_checkcif(1-114)/Ag3Pz3_110/Ag3Pz3_110_checkcif.pdf]
